# Using Gene Expression Analysis to Examine Changes in Loneliness, Depression and Systemic Inflammation in Lonely Chronically Ill Older Adults^*^

**DOI:** 10.4236/ojn.2016.68066

**Published:** 2016-08-31

**Authors:** Laurie A. Theeke, Jennifer A. Mallow, Julie Moore, Ann McBurney, Reyna VanGilder, Taura Barr, Elliott Theeke, Stephanie Rellick, Ashley Petrone

**Affiliations:** 1 School of Nursing, West Virginia University, Morgantown, WV, USA; 2 Department of Family Medicine, West Virginia University, Morgantown, WV, USA

**Keywords:** Loneliness, Depression, Gene Expression, Inflammation

## Abstract

**Purpose:**

The purpose of this study was to evaluate the effectiveness of LISTEN (Loneliness Intervention) on loneliness, depression, physical health, systemic inflammation, and genomic expression in a sample of lonely, chronically ill, older adults.

**Methods:**

This was a prospective, longitudinal randomized trial of LISTEN, a novel intervention based on theories of narrative and cognitive restructuring to target specific aspects of loneliness. Twenty-three older, lonely, chronically ill adults were recruited from a family medicine clinic in West Virginia. Participants were randomized to two groups, 13 in LISTEN group (Loneliness Intervention) and 10 in attention control (healthy aging education). Participants attended an enrollment session where they completed consent, survey data (including sociodemographics and chronic illness diagnoses), baseline physical measures, and blood sampling for gene expression analysis. After completing the 5 weekly sessions, all participants attended a 12 week post data collection meeting (17 weeks post-baseline) for survey completion, physical measures and blood sampling.

**Results:**

The results of this study show that the LISTEN intervention improves measures of physical and psychosocial health. Specifically, subjects enrolled in LISTEN showed reductions in systolic blood pressure, as well as decreased feelings of loneliness and depression. These changes may be due, in part, to a reduction in systemic inflammation, as measured by interleukin-2.

**Conclusion:**

This study provides support for the use of LISTEN in reducing loneliness in chronically ill, older adults. Further, while some of our results are inconclusive, it provides rationale to expand our study population to evaluate the relationship between loneliness and systemic inflammation. In the future, enhancing knowledge about the relationships among loneliness, chronic illness, systemic inflammation, and gene expression of these particular targets, and how these relationships may change over time with intervention will inform translation of findings to clinical settings.

## 1. Introduction

Loneliness is a biopsychosocial stressor that elicits a neuroimmunological stress response that has been consistently associated with multiple cardiovascular morbidities like hypertension [1]–[3] and coronary artery disease [4] [5], immunosuppression [6]–[8], elevated blood glucose [9], and depression [10] [11]. The exact mechanism by which loneliness affects physical health is unclear but it is thought to be mediated through immune [12]–[14] and inflammatory regulation over time [1] [4] [15]. Recent studies indicate that socially stressed persons are at increased risk for other chronic inflammation-related illnesses like neurodegenerative diseases [16] [17] and some cancers [18] [19].

Interleukin-2 (IL-2) and interleukin 6 (IL-6) are two commonly used markers of inflammation, and increased expression of both IL-2 and IL-6 has been shown in cardiovascular disease [20], COPD [21] [22], loneliness [23]–[25] and depression [26] [27]. Pro-inflammatory cytokines, such as IL-2 and IL-6, act on the brain by activating the hypothalamic-pituitary-adrenal (HPA) axis. HPA axis activation ultimately leads to a secretion of glucocorticoids from the adrenal cortex, mainly cortisol, leading to inhibition of pro-inflammatory signaling pathways [16] [18]. The dynamics between inflammation, HPA-axis activation, and loneliness are quite complex. The majority of studies suggest that loneliness is associated with both increase inflammation and increased cortisol secretion, but given the negative feedback regulation of cortisol on pro-inflammatory signaling, these findings together may seem contradictory [28]. As such, there is still much to be known about the dynamics of inflammation and HPA-axis activity, especially in regards to loneliness.

Understanding the role of IL-6 in the inflammatory process is difficult since decreases in Il-6 may actually be a sign of failing to self-regulate an inflammatory process [29]. One gene expression study of 25 participants in the top quartile of the UCLA Loneliness scale scores over a 3 year period, controlled for age, gender, race/ethnicity, marital status, income, and BMI, reports that 98 genes show a ≥ 15% difference in average expression in high-lonely individuals when compared to low lonely people. In this study, highly lonely people have a repressive effect on leukocyte activation and it is important to note in this study that the high lonely people have a mean score of 46.5 on the UCLA loneliness scale (scale range 20 – 80) [8].

The purpose of this study was to evaluate the effectiveness of LISTEN (Loneliness Intervention) on loneliness, depression, physical health, systemic inflammation, and genomic expression in a sample of lonely, chronically ill, older adults. The study had three aims: 1) to describe the relationship between chronic illness burden and loneliness; 2) to describe the changes in loneliness, depression, blood pressure, and BMI over time in chronically ill lonely older adults who have participated in a randomized trial of an intervention for loneliness, and 3) to describe differences in the systemic inflammation and gene expression between participants of LISTEN and attention-control groups.

## 2. Methods

### 2.1. Human Subjects Protection

This study was approved by the WVU Institutional Review Board. Written informed consent was obtained from all subjects or their authorized representatives prior to performing study procedures.

### 2.2. Design, Sample, and Setting

**Figure 1:**
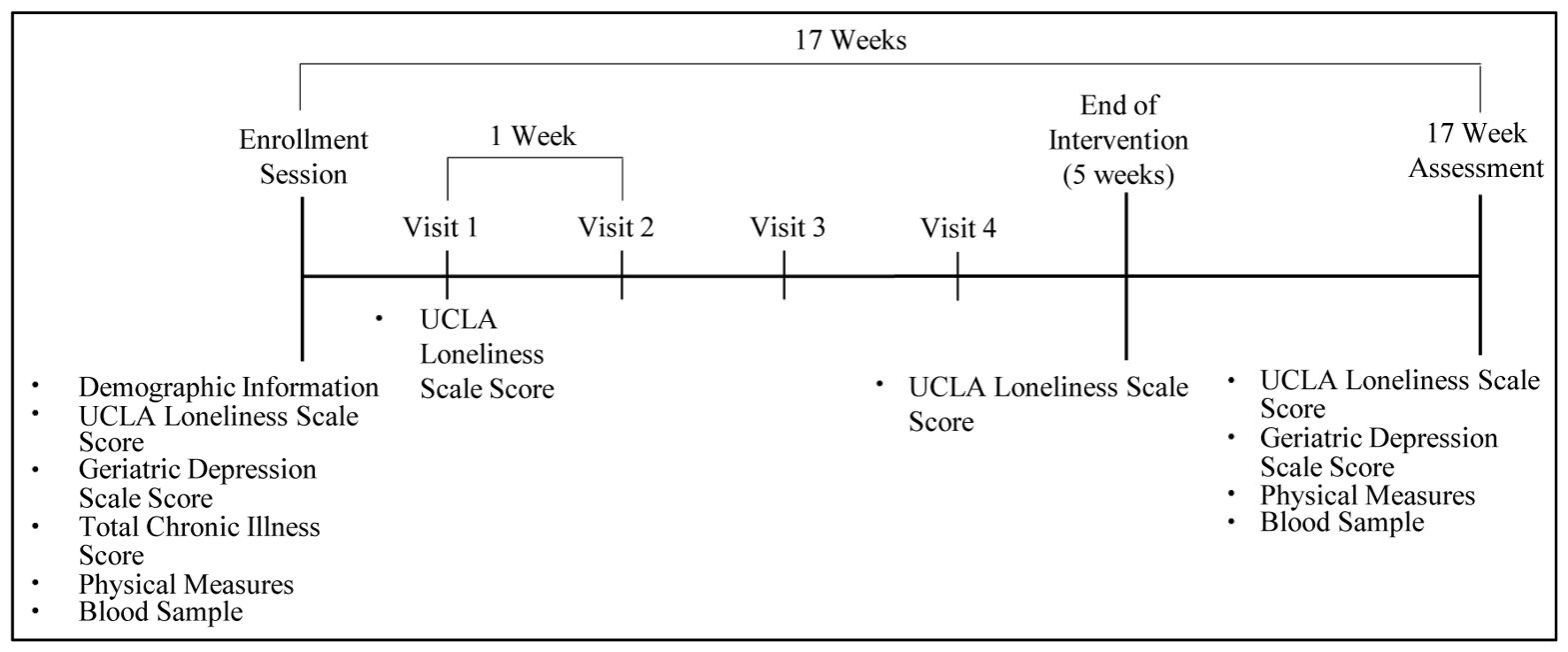
LISTEN trial study design.

This was a prospective, longitudinal randomized trial of LISTEN, a novel intervention designed to target specific aspects of loneliness. LISTEN is based on theories of narrative and principles of cognitive restructuring. The development of LISTEN and initial feasibility and acceptability has been previously published in OJN [30] [31]. Twenty-three older, lonely, chronically ill adults were recruited from a primary care center at a university based family medicine clinic in West Virginia between September 2013 and September 2015. To be included, participants had to be aged 65 years or older, living in the community, chronically ill, able to interact in a group intervention setting (minimum mini-mental status score of 23), and moderately lonely (a minimum score of 40 on the revised UCLA Loneliness Scale) [32]. Participants were randomized to two groups, 13 in LISTEN group (Loneliness Intervention) and 10 in attention control (AC) (healthy aging education). Participants of LISTEN attended sessions that focused on belongingness, relationships, socialization in community, challenges of loneliness, and meaning of loneliness. Participants of the attention control groups received educational lectures on physical changes associated with aging, healthy diet, aging health, prevention of stroke, and routine preventive healthcare. All participants attended 5 sequential weekly 2 hour sessions in the same building at the same times on the same days to ensure fidelity. After completing the 5 weekly sessions, all participants attended a 12 week post data collection meeting (17 weeks post-baseline) for survey completion, physical measures and blood sampling (Figure 1). All sessions were audio and video recorded.

Participants attended an enrollment session where they completed consent, survey data (including sociodemographics and chronic illness diagnoses), baseline physical measures, and blood sampling. Sociodemographic information collected included age, gender, marital status, highest level of education completed, household income, and employment status. Physical measurements included height, weight, blood pressure, salivary cortisol, and number of chronic illness diagnoses. Psychosocial measures included depression via the 5 item Geriatric Depression Scale (GDS) [33] and loneliness via the revised UCLA Loneliness scale [32]. The development of the 5-item GDS was based on the diagnostic criteria from the Diagnostic and Statistical Manual of Mental Disorders, Fourth Edition. Scores on this scale range from 0 – 5 and the scale has a reported sensitivity of 0.94 and a specificity of 0.81 [33]. The UCLA Loneliness scale scores range from 20 – 80, with 80 indicating very high loneliness.

### 2.3. Measuring Salivary Cortisol Levels

Salivary cortisol concentrations were measured using the Adrenocortex Stress Profile kit (Genova Diagnostics). Per the kit manufacturer instructions, saliva was collected at four time points to allow for circadian patterns of cortisol release: morning within one hour of waking, midday (11 am – 1 pm), afternoon (3 pm – 5 pm), and evening (10 pm – 12 pm).

### 2.4. Blood Sample Collection

Whole blood samples were drawn into PAXgene tubes during standard phlebotomy. PAXgene tubes were immediately inverted 8 – 10 times to ensure red blood cell lysis, de-identified and stored for future analysis. The PAXgene blood RNA tubes contain a reagent that prevents RNA degradation and preserves the RNA expression profile. The stored specimens were assigned a number that is unrelated to any patient identifiers. The blood samples were stored in a −80°C freezer until the analysis was conducted.

### 2.5. RNA Extraction

PAXgene^®^ Blood RNA tubes were thawed overnight (16 – 20 h) at room temperature prior to RNA extraction. The PAXgene Blood RNA kit (Pre-Analytix) was used to purify/extract intracellular RNA, per manufacturer’s instructions. RNA concentration and quality was determined by absorbance using a Take3 Trio Microplate (BioTek^®^) read on a Syntek Hybrid Plate Reader and analyzed using Gen5 (BioTek^®^) software. A260/A280 values between 1.8 and 2.2 were considered acceptable RNA quality.

### 2.6. Gene Expression Analysis

RNA was converted to complementary DNA (cDNA) using the High-Capacity Reverse Transcription Kit (Applied Biosystems). cDNA (10 ng) was used for quantitative real-time polymerase chain reaction (PCR) amplification using Taqman^®^ gene expression probes (Applied Biosystems) on the Step One Real-Time PCR system (Applied Biosystems). Taqman^®^ probes for *IL*-2 and *IL*-6 were used to detect gene expression samples, and relative expression of *IL*-2 and *IL*-6 was normalized to the expression of the reference gene, Beta-Actin. Fold change differences between groups for *IL*-2 and *IL*-6 were calculated by the ΔΔCT method [34].

### 2.7. Data Analysis

All statistical analyses were performed using IBM SPSS Statistics (Version 24). Statistical significance was taken at the 5% alpha level (*p* < 0.05). Fisher’s exact test to compare AC and LISTEN groups for categorical variables, such as gender, marital status, education, and household income.

#### Analysis of Aim 1

Spearman rank correlation was used to measure the association between chronic illness burden, UCLA loneliness scale score, and GDS. Mann-Whitney U test was used to compare UCLA loneliness scale score and GDS between AC and LISTEN groups.

#### Analysis of Aim 2

Spearman rank correlation was used to measure the association between physical measurements, such as blood pressure and BMI. Mann-Whitney U test was used to compare physical measurements between AC and LISTEN groups.

#### Analysis of Aim 3

Mann-Whitney U test was used to measure differences in *IL*2 and *IL*6 expression between AC and LISTEN groups. Fold changes were calculated using the ΔΔCT method as described above, and standard deviation of fold changes was calculated using ΔCT values. Mann-Whitney U test was also used to measure differences in salivary cortisol concentrations between AC and LISTEN groups.

## 3. Results

A total of 23 participants completed this study (N = 10 AC, N = 13 LISTEN). The baseline characteristics of participants by group are summarized in Table 1. There were no significant differences between groups for demographics, physical measurements, or psychosocial assessments at baseline.

### 3.1. The Relationship between Chronic Illness, Loneliness, and Depression

**Figure 2:**
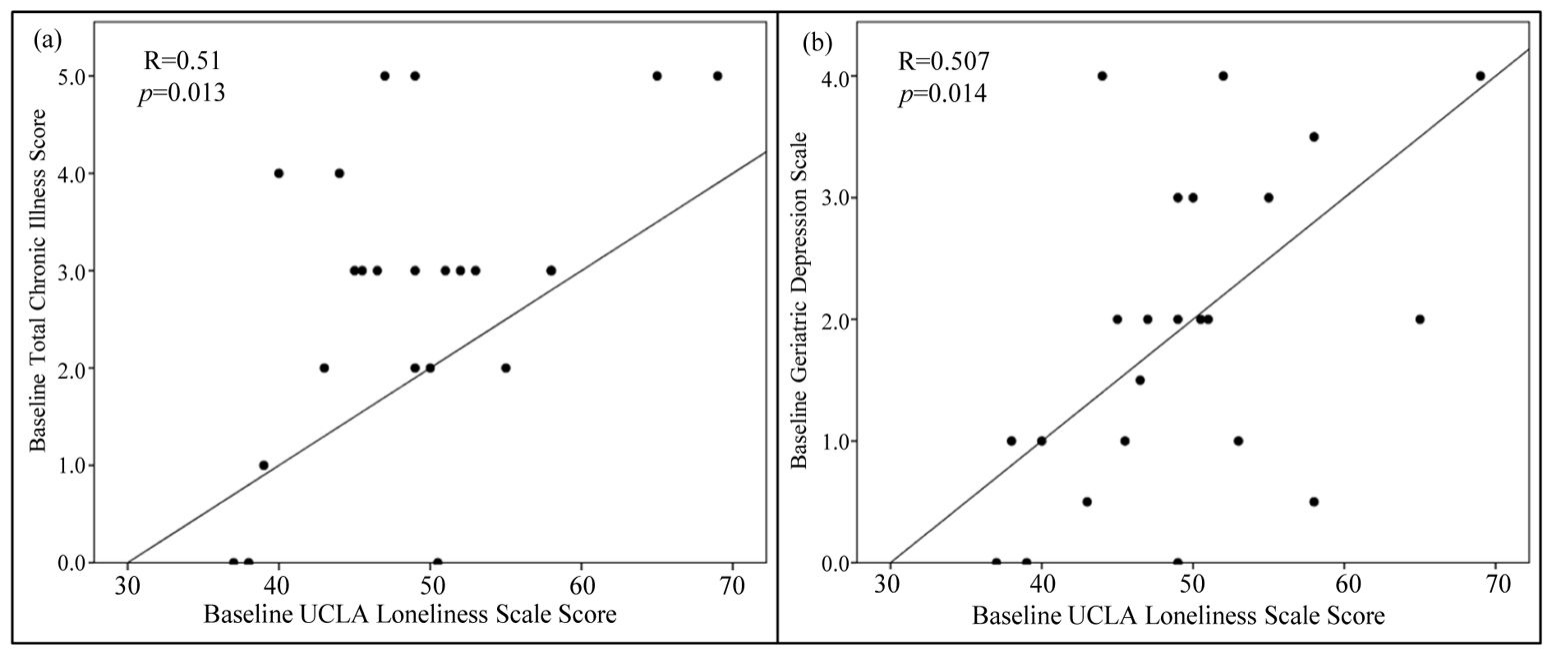
The relationship between chronic illness, loneliness, and depression. (a) There is a positive correlation between total chronic illness score and scores on the UCLA loneliness scale at baseline (r = 0.51, p = 0.013). (b) There is a positive correlation between scores on the UCLA loneliness scale and Geriatric Depression Scale at baseline (r = 0.507, p = 0.014).

There is a positive correlation between total chronic illness score and scores on the UCLA loneliness scale at baseline (r = 0.51, p = 0.013, supporting the premise that subjects with a higher burden of chronic illness tend to be more lonely (Figure 2(a)). Further, there is a positive correlation between scores on the UCLA loneliness scale and Geriatric Depression Scale at baseline(r = 0.507, p = 0.014) (Figure 2(b)), and at 17 weeks (r = 0.453, p = 0.03) (not shown).

### 3.2. The Effect of LISTEN on Loneliness and Depression

**Figure 3:**
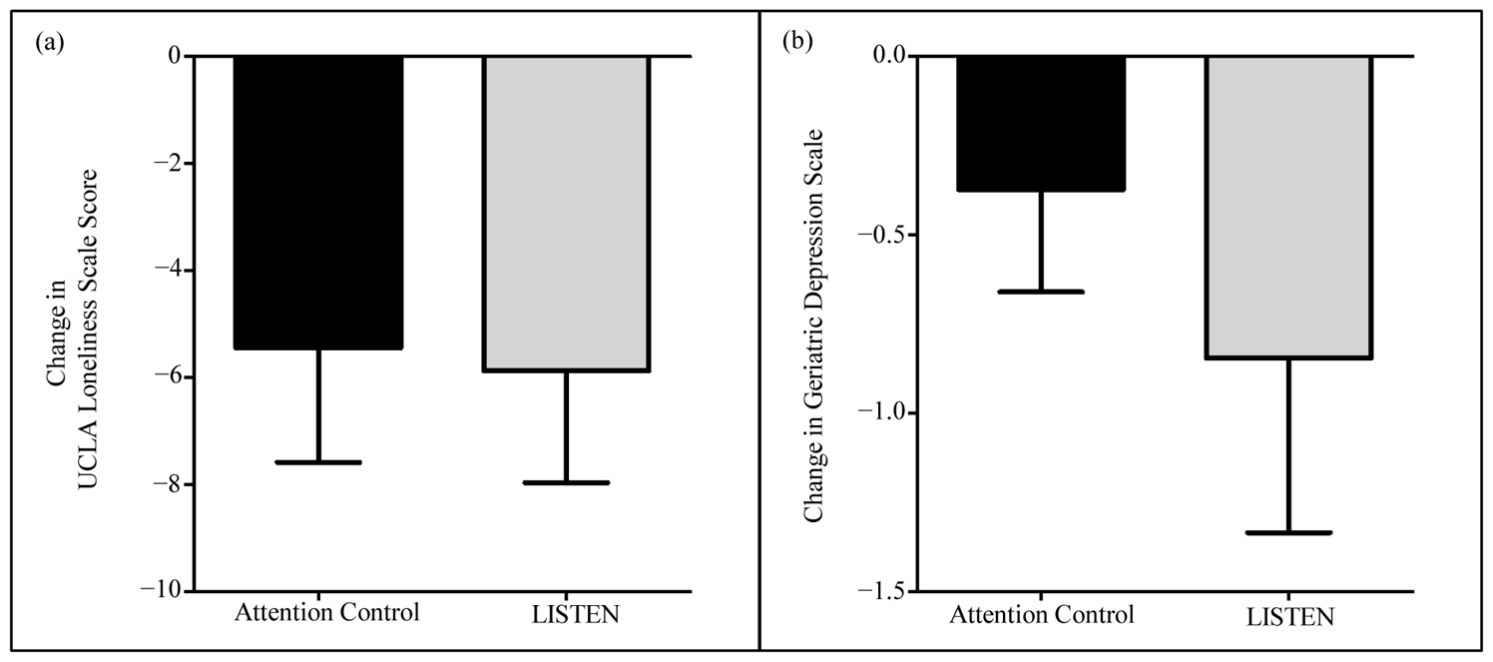
The effect of listen on loneliness, and depression. (a) UCLA Loneliness Scale Score was more greatly reduced in the LISTEN compared to attention control (AC = −4.9 ± 6.3, LISTEN = −5.4 ± 7.15, p = 0.856). (b) Geriatric Depression Scale Score was more greatly reduced in the LISTEN compared to attention control; however, this reduction did not reach statistical significance (p = 0.448).

**Figure 4:**
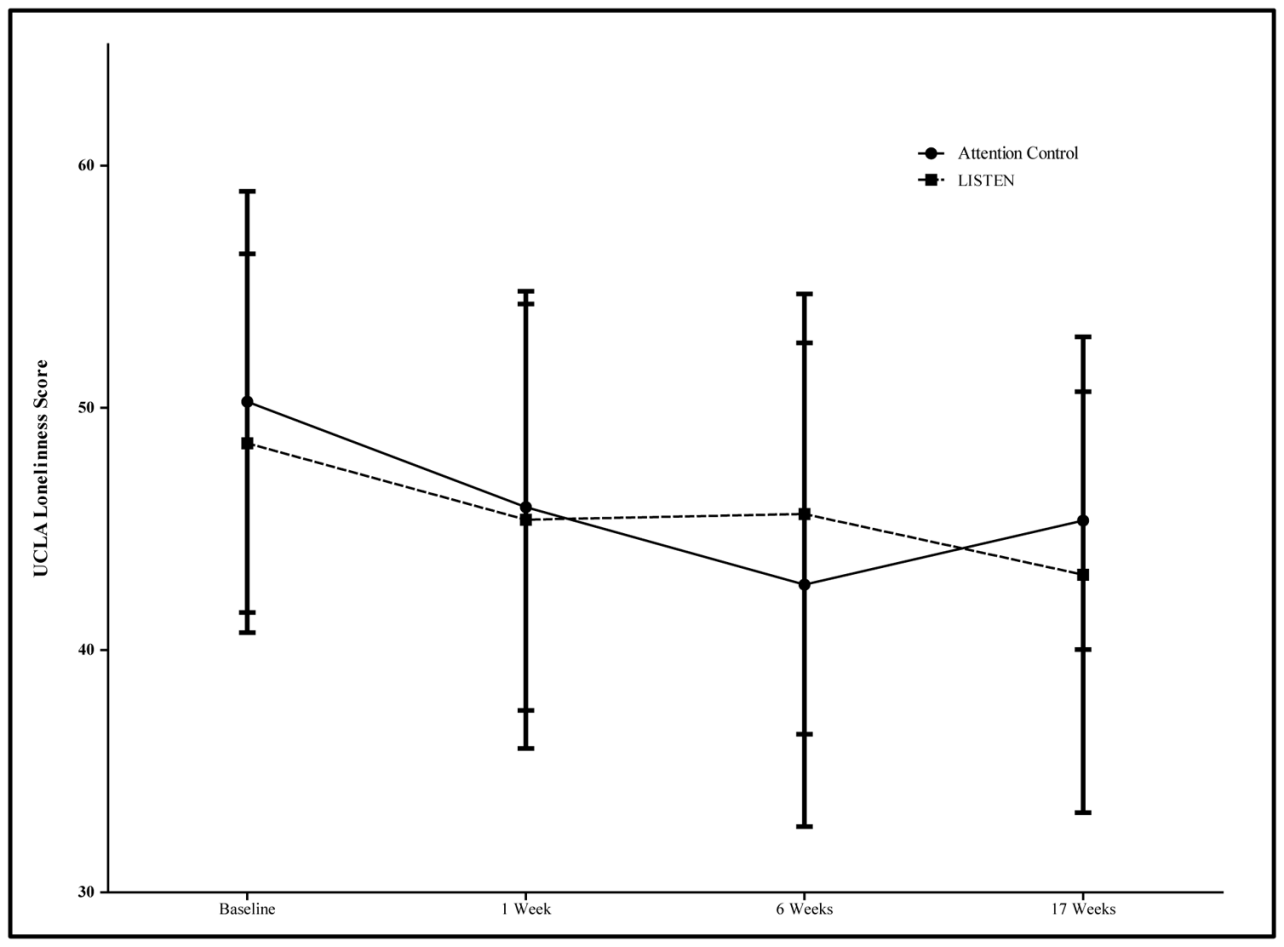
The effect of LISTEN on loneliness across study period (UCLA Loneliness Scale Score at each time point compared between attention control and LISTEN group). There was an increase in loneliness in the AC group (mean change = 3 ± 7.33), whereas loneliness continued to decrease from 6 to 17 weeks in the LISTEN group (mean change = −0.25 ± 6.39) (p = 0.067).

While subjects enrolled in the LISTEN group appeared to have a greater decrease in UCLA loneliness scores across 17 weeks compared to subjects in the AC group, this reduction was not statistically significant (AC = −4.9 ± 6.3, LISTEN = −5.4 ± 7.15, p = 0.856) (Figure 3(a)). There was also a decrease in Geriatric Depression Scale Score in both the AC and the LISTEN group; however, this decrease was not statistically significant within either group (AC-p = 0.229, LISTEN-p = 0.109), nor between groups (p = 0.448) (Figure 3(b)). Interestingly, while the change in UCLA loneliness scores from baseline to 17 weeks did not differ dramatically between groups, the change in UCLA loneliness scores from 6 weeks (end of intervention) to 17 weeks were more significant. Specifically, there was an increase in loneliness in the AC group (mean change = 3 ± 7.33), whereas loneliness continued to decrease from 6 to 17 weeks in the LISTEN group (mean change = −0.25 ± 6.39) (p = 0.067) (Figure 4). We observed no significant changes in neither daily average salivary cortisol levels nor diurnal decline in salivary cortisol between the AC and LISTEN groups at any time point, nor within each of the groups from baseline to 17 weeks. Table 2 summarizes the salivary cortisol concentrations at each time point for the groups.

### 3.3. The Impact of LISTEN on Physical Health Measures

**Figure 5:**
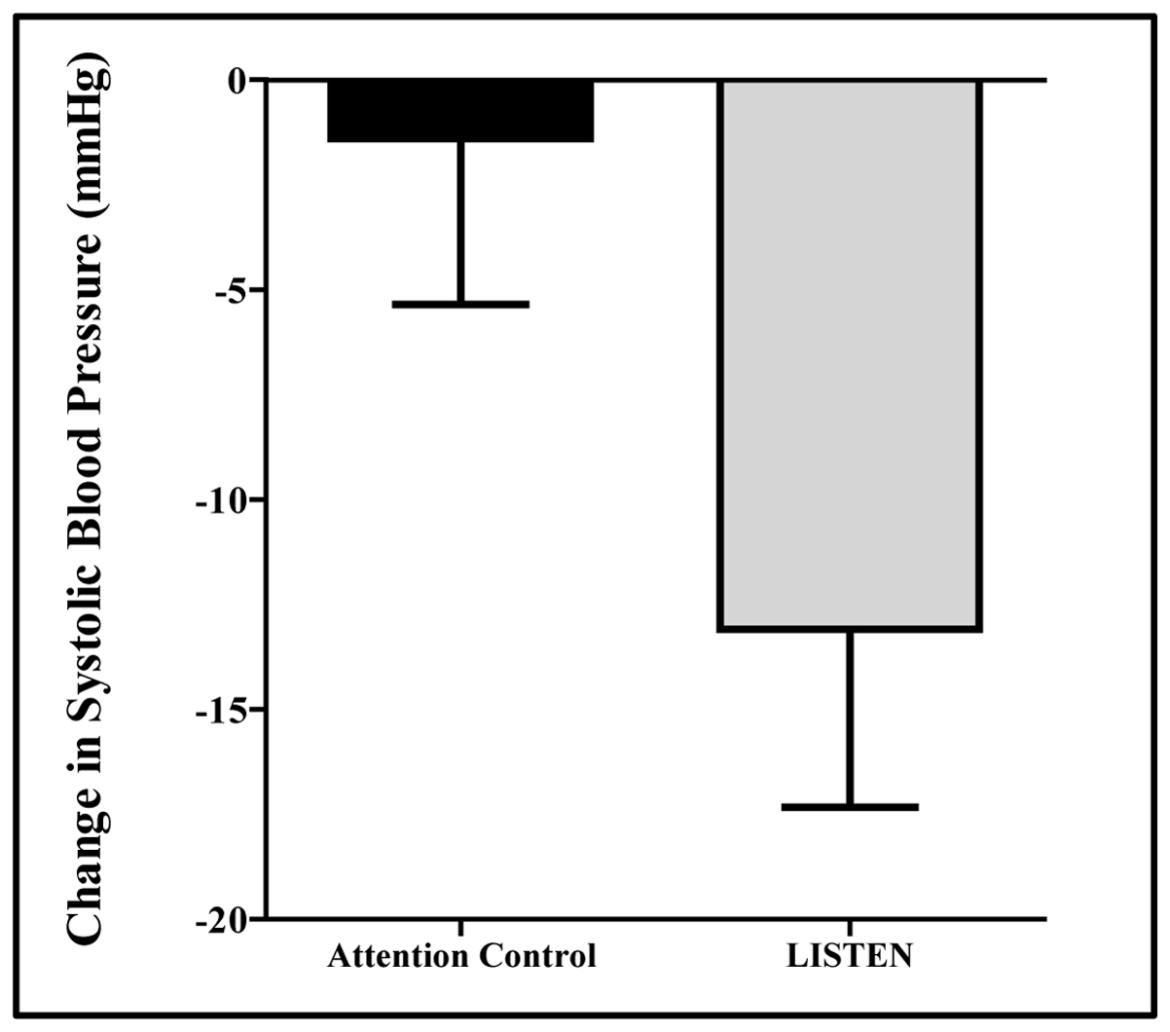
The effect of LISTEN on systolic blood pressure (systolic blood pressure at 17 weeks compared between attention control and LISTEN group). There was a considerable reduction in systolic blood pressure in the LISTEN group compared to the AC group at 17 weeks (no intervention = −1.4 ± 12.5, LISTEN = −11.1 ± 13.8, p = 0.013).

There was a considerable reduction in systolic blood pressure in the LISTEN group compared to the AC group at 17 weeks (no intervention = −1.4 ± 12.5, LISTEN = −11.1 ± 13.8, p = 0.013) (Figure 5). There were no significant changes in diastolic blood pressure in either group. Further, this decrease in systolic blood pressure was correlated with a decrease in BMI (r = 0.552, p = 0.008) (not shown). While subjects in both the AC and the LISTEN group showed a decrease in BMI, this decrease was not statistically significant in either group (AC-p = 0.251, LISTEN-p = 0.498), nor between groups (p = 0.396) (not shown).

### 3.4. The Effect of LISTEN on *IL*2 and *IL*6 Expression

**Figure 6:**
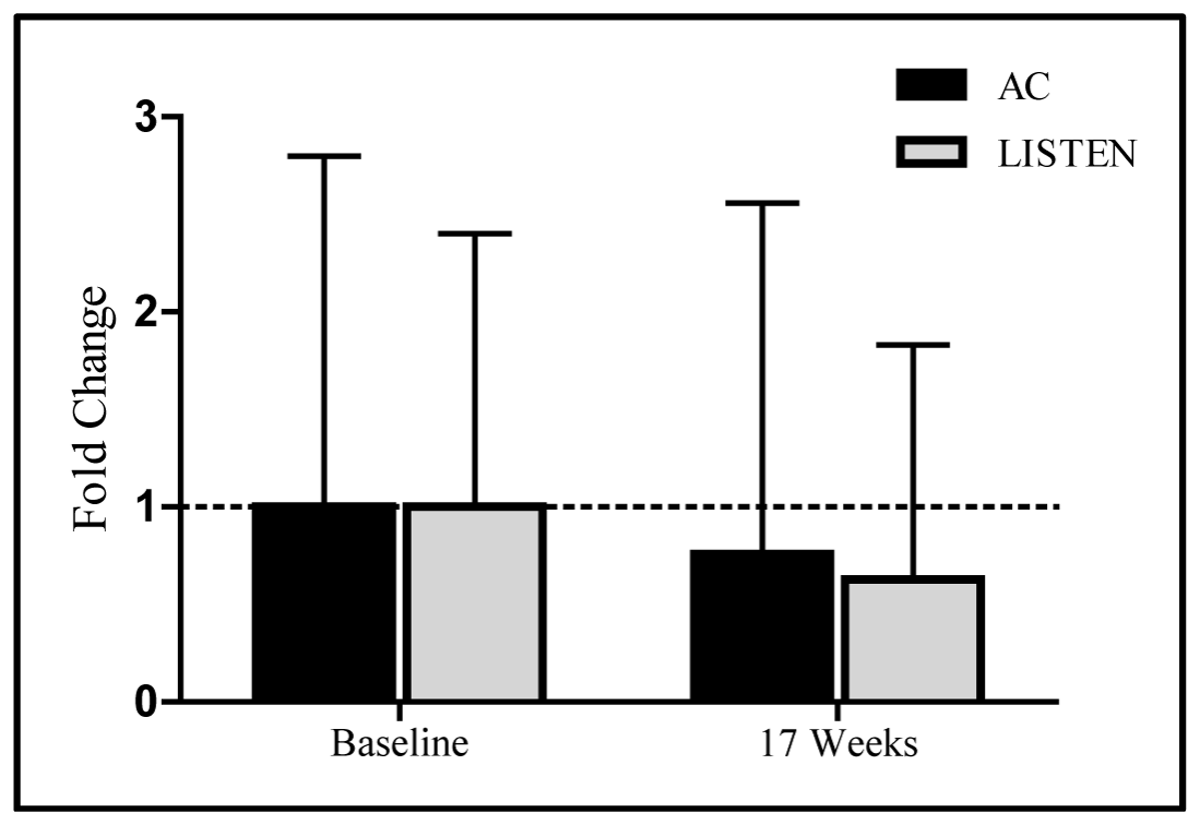
The effect of LISTEN on interleukin-2 mRNA expression (interleukin-2 mRNA expression fold changes from baseline to 17 weeks compared between attention control and LISTEN group). Subjects enrolled in the LISTEN group had a greater decrease in IL2 expression at 17 weeks compared to subjects who attended attention control group sessions but this reduction was not statistically significant (AC IL2 Fold Change = 0.8 ± 1.8, LISTEN IL2 Fold Change = 0.7 ± 1.2, p = 0.585).

Subjects enrolled in the LISTEN group had a greater decrease in *IL*2 expression at 17 weeks compared to subjects who attended attention control group sessions but this reduction was not statistically significant (AC *IL*2 Fold Change = 0.8 ± 1.8, LISTEN *IL*2 Fold Change = 0.7 ± 1.2, p = 0.585) (Figure 6). Neither subjects enrolled in LISTEN groups nor attention control showed a change in *IL*6 expression at 17 weeks (AC *IL*6 Fold Change = 1.2 ± 1.3, LISTEN *IL*2 Fold Change = 1.3 ± 1.1, p = 0.731) (not shown).

## 4. Discussion

The findings of this study on older adults who are living in Appalachia are similar to findings reported from national data [35] and prior studies on the relationship between loneliness and chronic illness [36]. Our pilot data previously revealed that, in a sample of 60 rural older adults, none were screened for loneliness but 33% were moderately lonely with prevalent multiple chronic conditions. National data analyses indicate that a higher total number of chronic illnesses are predictive of loneliness [37]. The finding that loneliness scores are correlated with depressive symptoms is consistent with the health and social science literature which identifies loneliness to be a unique predictor of depression [38].

Loneliness has been identified as an independent predictor of hypertension [39] through the physiological stress response. Logically leading to the conclusion that diminishing loneliness may diminish psychosocial stress and lead to diminished blood pressure. This is clinically important because controlling blood pressure decreases the likelihood of poor health outcomes such as stroke, heart disease, and metabolic syndrome [40].

Findings from this study indicated that both LISTEN and attention control groups decreased in weight and BMI. While not statistically significant, this is a short trial which makes the findings clinically important. It has been reported that lonely adults in varied populations experience obesity [41] and metabolic syndrome [42]. Poor health consequences of obesity and metabolic syndrome include multiple chronic conditions that are well-documented and very costly to the healthcare system. Thereby, understanding how psychosocial problems like loneliness relate to these conditions is imperative.

When considering the findings regarding systemic inflammation and gene expression, the findings are inconclusive. It was interesting that participants who did NOT receive LISTEN had a decrease in IL2 expression which could be interpreted to indicate diminished immunity over time as they continued to be lonely. Diminished immunity has been associated with a chronic lonely state [8]. However, since both groups did diminish in loneliness over the relatively short study and the LISTEN group also decreased in IL2 expression, the results were not definitive. Though IL6 trended up for both groups, this increase in Il-6 with intervention could be interpreted in multiple ways. Again, since both groups diminished in their loneliness scores, it could be that less lonely persons were better able to moderate inflammation. This would be consistent with recent studies reporting that higher loneliness is related to higher inflammation on measures such as C-reactive protein [43].

### 4.1. Future Research Implications

A better research approach should include a comprehensive assessment of the various arms of the immune system, not just single markers, across genomic and proteomic expression. This approach would require significant blood sampling/processing and a larger sample size than what we have studied here. The power of the techniques used for gene expression in this study lies in multiple sampling analyses and comparing the data over time amongst individuals. Future studies should be designed to optimize the technique as well as the biomarkers to be studied.

### 4.2. Conclusion

Loneliness is a prevalent problem that contributes to multiple chronic conditions, functional decline [44], and mortality [45] in older adults. Further, loneliness negatively impacts the healthcare system as lonely older adults are more likely to enter a nursing home [46], more frequently access primary care [47], have increased emergency care visits [48], and report increased use of formal support services [49]; making loneliness a priority for study in this population. Although our results are not significant, we do observe trends that will inform future studies to further assess the inflammatory/immune patterns associated with loneliness. In the future, enhancing knowledge about the relationships among loneliness, chronic illness, systemic inflammation, and gene expression of these particular targets, and how these relationships may change over time with intervention will inform translation of findings to clinical settings.

## Figures and Tables

**Table 1:** Baseline demographic, physical, and psychosocial characteristics of study participants (N = 23)

Characteristic	LISTEN Group (N = 13)	Attention Control (N = 10)	Difference Statistic
**Demographic**			
Age (mean ± SD years)	75 ± 7.5	75 ± 8.8	*t* = 0.015, p *=* 0.98
Gender (% female)	85%	100%	*χ*^2^ = 1.69, p = 0.19
Marital Status (% married)	38%	20%	*χ*^2^ = 2.58, p = 0.46
Highest Education Completed (N (%))			*χ*^2^ = 4.35, p = 0.50
High School or Less	2 (15)	4 (40)	
Some College	6 (46)	2 (20)	
Undergraduate Degree	3 (23)	1 (10)	
Graduate Degree	2 (15)	3 (30)	
Household Income (N (%))			*χ*^2^ = 8.9, p = 0.26
Less than $20,000	3 (23)	6 (60)	
$20,001 – $30,000	4 (31)	2 (20)	
Over $30,000	6 (46)	2 (20)	
Employment Status (N (%))			*χ*^2^ = 0.21, p = 0.90
Retired and Not Working	10 (77)	7 (70)	
Working Part-Time	3 (23)	3 (30)	
**Physical**			
Body Mass Index (mean ± SD kg/m^2^)	31 ± 8.1	28 ± 7.5	*t =* −0.654, p *=* 0.52
Systolic Blood Pressure (mean ± SD mmHg)	140 ± 11	128 ± 16	*t* = −2.04, p *=* 0.07
Diastolic Blood Pressure (mean ± SD mmHg)	79 ± 7	75 ± 11	*t* = −1.16, p *=* 0.29
Total Chronic Illnesses (mean ± SD), (N (%))	3.1 ± 1.5	2.4 ± 1.6	*t* = −1.05, p *=* 0.31
Arthritis	8 (62)	7 (70)	
Cancer	3 (23)	3 (30)	
Diabetes	5 (38)	4 (40)	
Heart Disease	5 (38)	1 (10)	
Hypertension	10 (77)	3 (30)	
Lung Disease	5 (38)	4 (40)	
Stroke	1 (8)	0 (0)	
**Psychosocial**			
UCLA Loneliness Scale Score (mean ± SD)	48.5 ± 7.8	50.3 ± 8.7	*t =* 0.49, p *=* 0.625
Geriatric Depression Scale Score (mean ± SD)	2.04 ± 1.3	1.65 ± 1.4	*t =* −0.70, p *=* 0.493

**Table 2:** Salivary cortisol levels in attention control versus LISTEN participants (mean ± SD ug/dL)

	Wake (7 am – 9 am)	11 am – 1 pm	3 pm – 5 pm	Bedtime (10 pm – 12 am)	Diurnal Slope
**AC**					
Baseline	0.7 ± 0.4	0.3 ± 0.1	0.3 ± 0.2	0.1 ± 0.05	−0.6 ± 0.4
12 Weeks	0.5 ± 0.2	0.4 ± 0.3	0.3 ± 0.1	0.2 ± 0.1	−0.4 ± 0.2
**LISTEN**					
Baseline	0.7 ± 0.5	0.2 ± 0.1	0.3 ± 0.08	0.1 ± 0.05	−0.6 ± 0.5
12 Weeks	0.7 ± 0.6	0.3 ± 0.3	0.2 ± 0.2	0.2 ± 0.3	−0.5 ± 0.7
